# Impairment of Autophagic Flux Promotes Glucose Reperfusion-Induced Neuro2A Cell Death after Glucose Deprivation

**DOI:** 10.1371/journal.pone.0076466

**Published:** 2013-10-04

**Authors:** Bong Geom Jang, Bo Young Choi, Jin Hee Kim, Min-Ju Kim, Min Sohn, Sang Won Suh

**Affiliations:** 1 Department of Physiology, Hallym University, College of Medicine, Chuncheon, Republic of Korea; 2 Department of Anatomy and Neurobiology, Hallym University, College of Medicine, Chuncheon, Republic of Korea; 3 Inha University, Department of Nursing, Incheon, Republic of Korea; China Medical University, Taiwan

## Abstract

Hypoglycemia-induced brain injury is a common and serious complication of intensive insulin therapy experienced by Type 1 diabetic patients. We previously reported that hypoglycemic neuronal death is triggered by glucose reperfusion after hypoglycemia rather than as a simple result of glucose deprivation. However, the precise mechanism of neuronal death initiated by glucose reperfusion is still unclear. Autophagy is a self-degradation process that acts through a lysosome-mediated trafficking pathway to degrade and recycle intracellular components, thereby regulating metabolism and energy production. Recent studies suggest that autophagic and lysosomal dysfunction leads to abnormal protein degradation and deposition that may contribute to neuronal death. Here, we focused on the relationship between autophagy and lysosomal dysfunction in hypoglycemia-induced neuronal death. In neuronal cells, glucose reperfusion after glucose deprivation resulted in inhibition of autophagy, which may promote cell death. This cell death was accompanied with activation of caspase3 and the lysosomal proteases cathepsin B and D, which indicated impairment of autophagic flux. Taken together, these results suggest that interplay of autophagy, caspase3 activation and lysosomal proteases serve as a basis for neuronal death after hypoglycemia. Thus, we provide the molecular mechanism of neuronal death by glucose reperfusion and suggest some clues for therapeutic strategies to prevent hypoglycemia-induced neuronal death.

## Introduction

Hypoglycemia, known commonly as “low blood glucose” or “low blood sugar”, is a state characterized by an abnormally low level of blood glucose compared with the normal physiologic range. The most common form of hypoglycemia occurs as a complication in diabetic patients who attempt tight control of blood glucose levels with insulin or oral glucose lowering medications [1]. Glucose is a major metabolic fuel for the brain, which cannot synthesize glucose; therefore an insufficient supply of glucose to the brain results in a loss of neurons as well as impairment of function [2]. According to studies using animal models, acute/severe hypoglycemia [blood glucose (BG) < 18 mg/dL; 1 mM/L] induces neuronal damage in the vulnerable neurons of cortex and hippocampus [3]. In particular, this neuronal injury in hippocampus results in a decline in learning and memory [4]. Thus, understanding of the mechanisms of neuronal death accompanying hypoglycemia is fundamentally important for the prevention of post-hypoglycemia pathophysiology. Although hypoglycemic brain injury was first demonstrated by Auer three decades ago[3], little is known about the precise molecular mechanism of neuronal death by hypoglycemia.

We previously suggested that hypoglycemia-induced neuronal death is triggered by glucose reperfusion after acute/severe hypoglycemia rather than by hypoglycemia per se [5]. Accumulating evidence has demonstrated that glucose reperfusion injury is a multi-factorial process, ultimately culminating in hypoglycemia-induced neuronal death. For example, glucose reperfusion after hypoglycemia triggers activation of NADPH oxidase, which causes reactive oxygen species (ROS) production, subsequent activation of poly(ADP-ribose) polymerase, and resultant neuronal death [5]–[7]. Also, mitochondrial permeability transition and calpain activation have been shown to accompany hypoglycemia-induced neuronal death [8]. However, the precise molecular mechanism(s) that lead(s) to neuronal cell death by glucose reperfusion after hypoglycemia is still unclear.

Autophagy is a conserved catabolic process involving the degradation of intracellular macromolecules and organelles in mammalian cells via the lysosomal system. During autophagy, the cellular components are sequestered into double-membrane vesicles (autophagosomes), which then fuse with lysosomes, forming autolysosomes. These multiple, sequential processes are referred to as the autophagic flux. Subsequently, the breakdown products generated by hydrolytic enzymes in the lysosome are recycled for macromolecular synthesis and ATP generation. Autophagic flux can be monitored by measuring conversion of LC3I to LC3II and levels of substrates normally degraded by autophagy such as p62/SQSTM1 (SQSTM1 is sequestosome 1). The LC3 protein (microtubule-associated protein light-chain 3; also known as Atg8) is processed to LC3I in the cytosol and then recruited to autophagosome membranes as a phosphatidylethanolamine-conjugated form, LC3II [9]. Therefore, the levels of LC3II correlate with the number of autophagosomes [10]. The p62 protein directly binds to LC3, as well as to ubiqutinated substrates, and is degraded in autolysosomes [11]. Thus, increased levels of p62 are a reliable indicator of suppressed autophagy and increased autophagic flux is indicated by decreased p62 levels [12].

In neuron, autophagy is important for maintenance of cellular homeostatic functions such as quality control of proteins and organelles [13], [14]. Moreover, autophagy is essential for neuronal development as well as neuronal remodeling through regulation of axonal function and structure [15]–[17]. Thus basally or optimally induced autophagy is required to maintain cellular homeostasis and function in the neuron. Despite having an important role in maintaining health and integrity in neurons, the role of autophagy in survival and death is controversial [18], [19]. In many settings, autophagy has been shown to be neuroprotective effect but in some settings may promote neuronal cell death through abnormal degradation or deposition of cellular components [20]. For example, some studies have reported that defective basal autophagy or insufficient autophagy contributes to pathogenesis in neurodegenerative disease such as Parkinson disease, Alzheimer disease, and Huntington’s disease [14], [21] [22], [23]. On the other hand, additional studies suggest that excessively activated autophagy might contribute to neuronal death in various pathological conditions including cerebral ischemia [24].

Lysosomal dysfunction is closely related to impairment of autophagic flux [25]. Lysosomal overload or destabilization can lead to abnormal protein accumulation through inhibition of autophagosome fusion with the lysosome or defects the degrative functions of the lysosome. Similar to impairment of autophagy, dysfunction of lysosomal intracellular trafficking has also been linked to a variety of neurodegenerative diseases [26]. Lysosomes contain more than eighty hydrolytic enzymes such as cathepsins, nucleases, glycosidase, sulfatase and lipases. Cathepsins are major proteases of the lysosome and subdivide into three subgroups according to the amino acid sequence of the active site; the three subtypes are cysteine, aspartyl and serine cathepsins. In particular, cathepsin B, L and D are highly abundant in neurons. Lysosomal dysfunction can lead to release of these lysosomal proteases and subsequence cell death because the abnormal presence or activity of lysosomal proteases in the cytosol causes digestion of vital proteins and the activation of additional hydrolases including caspases [27].

The aim of this study is to elucidate the molecular mechanism underlying glucose reperfusion-induced neuronal death after hypoglycemia. Here, following glucose reperfusion, abnormal activities of autophagy, caspase3 and lysosomal protease, cathepsin B/D were observed in neuronal cell culture model. These results demonstrate not only a role for autophagy in glucose reperfusion injury, but also a possible link between autophagy, apoptosis and the lysosome in neuronal death in this setting. Finally, the present study suggests that interplay of autophagy, caspase3 and lysosomal proteases is a major driver of neuronal death by glucose reperfusion.

## Materials and Methods

### Antibodies, Reagents and Plasmids

Mouse monoclonal antibodies to p62 (Abnova,H00008878-M01), alpha-tubulin (Sigma,T6074), beta-actin (Sigma, A5316) and rabbit polyclonal antibodies to LC3 (Novus, NB100-2220), cleaved-caspase3 (Cell Signaling, #9664), Poly (ADP-ribose) polymerase (PARP, Cell Signaling, #9542) and goat polyclonal antibodies to cathepsin D (Santa Cruz, sc6486), cathepsin B (Santa Cruz Biotechnology, sc6493) were purchased from commercial sources as listed. 3-methyladenine (3MA, M9281), Pepstatin A (P5318), Leupeptin(L2884) were purchased from Sigma. Bafilomycin A1 (BA1, calbiochem,196000), pan-caspase inhibitor z-VAD-FMK (z-VAD, R&D system, FMK001) were purchased from commercial sources as listed. pEGFP–LC3 was provided by Addgene plasmid 21073 and mcherry-GFP-LC3 was a generous gift from Dr. Yong-Keun Jung (Seoul National University, Seoul, Korea).

### Cell culture and treatments

Mouse neuroblastoma cell line, neuro2A, was purchased from the American Type Culture Collection (ATCC). The neuro2A cells were cultured in Dulbecco’s modified Eagles medium (DMEM; GIBCO) supplemented with 10% (v/v) fetal bovine serum (FBS; GIBCO) at 37°C in a humidified atmosphere containing 5% CO_2_. For glucose deprivation/reperfusion, the neuro2A cells were plated at 50–60% confluence. On the following day, cells were pre-incubation in glucose-free DMEM medium (GIBCO) for 3 hour and then replaced by 20 mM glucose containing medium for 24 hour and 48 hour. For inhibition of autophagy, caspases or lysosomal protease, cells were treated with 3-methyldenine (3MA; 5 mM), Bafilomycin A1 (BA1; 1–5 nM), pan-caspase inhibitor z-VAD-FMK (z-VAD; 10 uM), leupeptin (Leu; 10 uM) or pepstatin (Pep; 10 uM) for 48 hour after 3 hour glucose deprivation.

### Cytotoxicity and viability assays

Neuro2a cells were plated on to 48-well plates at a density of 1.5×10^4 ^cells per well for LDH and MTT assay. Lactate dehydrogenase (LDH) release was measured using a cytotoxicity detection kit (TAKARA), according to the manufacturer’s protocol. After gentle agitation, 100 *µ*l of culture medium was collected at the noted times during the assay. Absorbance was measured at 405 nm using a 96-well microplate spectrophotometer (Thermo scientific). Cell viability was quantified by using MTT (3-(4,5-Dimethylthiazol-2-yl)-2,5-diphenyltetrazolium bromide, a yellow tetrazole, Sigma) reagent. MTT stock solution (5 mg/ml) was prepared in DPBS (GIBCO) and added to the culture media at a final concentration of 1 mg/ml. After 90 min incubation, the media was removed, and the chromogen in the cells was dissolved in DMSO containing 0.01 N NaOH. The absorbance at 570 nm was measured using a 96-well microplate spectrophotometer (Thermo scientific).

### Immunoblot analysis

Cells and tissuses were homogenized in RIPA buffer containing 10 mM Tris–HCl (pH 7.4), 150 mM NaCl, 1% Nonidet P-40, 0.5% sodium deoxycholate, 0.1% SDS. The homogenate was then centrifuged at 16,000 x g for 20 min at 4 °C. Protein concentration was determined using the bicinchoninic acid (BCA) assay (Sigma). Equal amounts of protein were separated by SDS–polyacrylamide gel electrophoresis (PAGE) and transferred onto the polyvinylidene fluoride membrane (PVDF, Millipore). After blocking, membranes were incubated with a primary antibody overnight at 4°C and then incubated with HRP-conjugated secondary antibodies (mouse and rabbit; Enzo, rat; Santa Cruz) for 1 hour at room temperature. Immunoreactivity was detected with enhanced chemoluminescent autoradiography (ECL kit, GE Healthcare), according to the manufacturer's instructions. Immunoreactivity was analyzed by densitometry using program Image J (NCBI) and was normalized to a loading control, alpha-tubulin or beta-actin.

### Transmission electron microscopy

To observe the ultrastructural changes in neuro2a cells, cells were subjected to GD for 3 hours followed by GR for 48 hours. Briefly, cells with 48 hour GR after 3hour GD were collected and compacted to solid pellets by centrifugation at 1200 x g for 3 min. The pellets were prefixed in 3% glutaraldehyde and postfixed in 2% osmium tetraoxide. After dehydration, they were embedded in epoxy resins. Ultrathin sections were cut, contrasted with uranyl acetate and lead citrate, and TEM analysis were performed on EF-TEM LEO 912AB (Carl Zeiss Inc., Germany, Korea Basic Science Institute, Chuncheon) operated at 120 kV.

### Transfection and LysoTracker staining

For GFP-LC3 or mCherry-GFP-LC3 detection, expression vectors were transfected into cells using the LIPOFECTAMINE™ 2000 reagent (Invitrogen) according to the manufacturer’s instructions. On the following day, the transfected cells were used for GD/GR examination and subsequent treatments of inhibitors. For LysoTracker staining, the transfected cells were washed with PBS and incubated in DMEM medium containing 1 mM LysoTracker Red DND-99 (Invitrogen) for 30 min. The cells were then washed twice with PBS and fixed in PBS containing 4% formaldehyde for 10 min at room temperature. Simultaneous detection of the LysoTracker and GFP-LC3 signal in neuro2a cells was performed as described for LysoTracker staining, using the endogenous fluorescent signal from GFP-LC3. Cells were evaluated under a fluorescence microscope (OlympusIX70; Olympus Corp.).

### Measurement of activities of cathepsin

Enzyme activity of cathepsin D or B was determined with cathepsin D or B activity fluorometric assay kit according to the manufacturer’s protocol (abcam). Briefly, cell lysates were centrifuged at 10,000×g for 10 min at 4°C and the supernatant was used for enzymatic assay. Protein was incubated at 37°C for 1–2 hours with GKPIFFRLK(Dnp)-DR-NH2-MCA (substrate for cathepsin D) or 10 mM Ac-RR-AFC (substrate for cathepsin B). After incubation for 1 hour, fluorescence was measured with a fluorescence microplate reader (SpectraMax Geminis XS; Molecular Devices, Inc.) at 328/460 nm (excitation/emission; for cathepsin D activity) or 400/505nm (excitation/emission; for cathepsin B activity).

### Statistical analysis

All data are expressed as mean values ± SD. Statistical significance was assessed by ANOVA and post hoc testing was performed using Scheffe’s test. For single comparisons, significance was evaluated with Student's *t*-test. *P*-values less than 0.05 were considered statistically significant.

## Results

### Glucose deprivation-induced neuronal cell death involves activation of autophagy and caspase3

**Figure 1:**
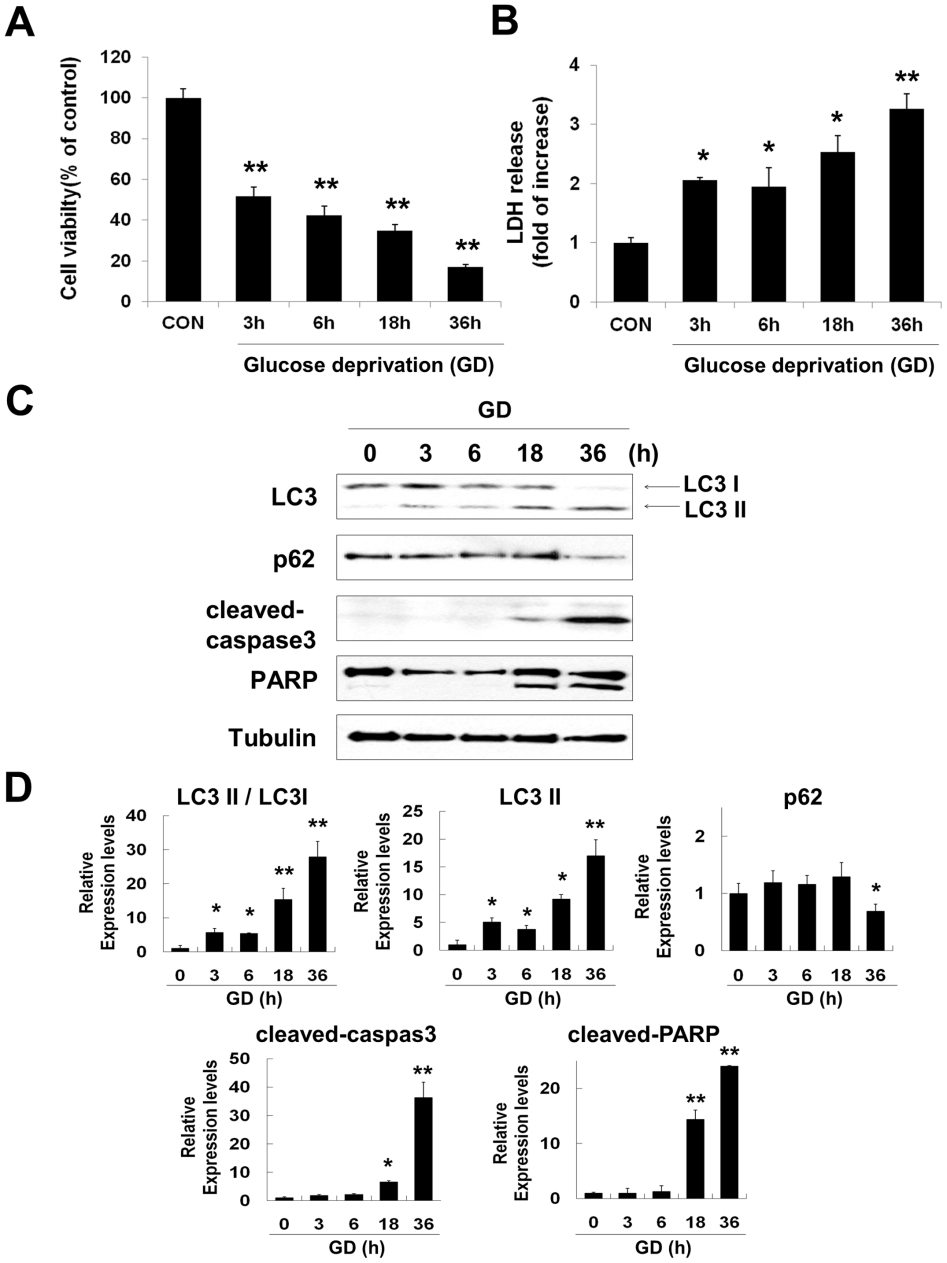
Glucose deprivation (GD) induces neuronal cell death. Time course of cell viability and cell death after GD was estimated by MTT and LDH assays, respectively. Bars depict rates of MTT reduction (A) and LDH release (B) time dependent manner after GD. MTT data represent relative percentages compared with controls (*p<0.05, **p<0.001, n = 3). LDH release from neuro2a cells was calculated as fold increase from the LDH release in controls (*p<0.01, **p<0.001, n = 3). (C) Levels of LC3 and p62 expression, cleaved-caspase 3 and PARP cleavage were determined at indicated times after GD by immunoblot analysis. Tubulin was used as loading control. (D) Quantitative analyses of the immunoblots are shown in (C). The amount of each protein was normalized against the amount of tubulin. Data represent the mean ± SD for each condition (*p<0.05, **p<0.001, n = 3).

To investigate the survival rate of neuronal cells under glucose deprivation (GD) conditions, MTT and LDH assays were performed for 3, 6, 18 and 36 h. GD caused time-dependent decrease in MTT reduction [indicated values represented by mean % : 100 (control), 51.8 (3 h), 42.3 (6 h), 34.7 (18 h), 17.2 (36 h)] (Fig. 1A) and an increase in LDH release [indicated values represented by fold of increase : 1 (control), 2.1 (3 h), 1.95 (6 h), 2.5 (18 h), 3.3 (36 h)] (Fig. 1B). These results demonstrated that GD caused a time-dependent decline in neuronal survival.

To determine whether autophagy and apoptosis were involved in GD-induced neuronal death, immunoblot analysis was performed: antibodies for LC3 and p62 were used for detection of autophagy and cleaved-caspase3 and PARP were used for detection of apoptosis (Fig. 1C). During GD, conversion of LC3I to LC3II increased in a time dependent manner and the level of p62 was decreased at 36h of GD (Fig, 1D). Markers of apoptosis, levels of cleaved-caspase3 and PARP cleavage, were detected after 18 h of GD and increased in a time dependent manner (Fig. 1D). These results show that activation of autophagy and apoptosis is involved in neuronal cell death during GD.

### Glucose reperfusion-induced neuronal death is associated with autophagic flux and caspase-3 dependent apoptosis

**Figure 2:**
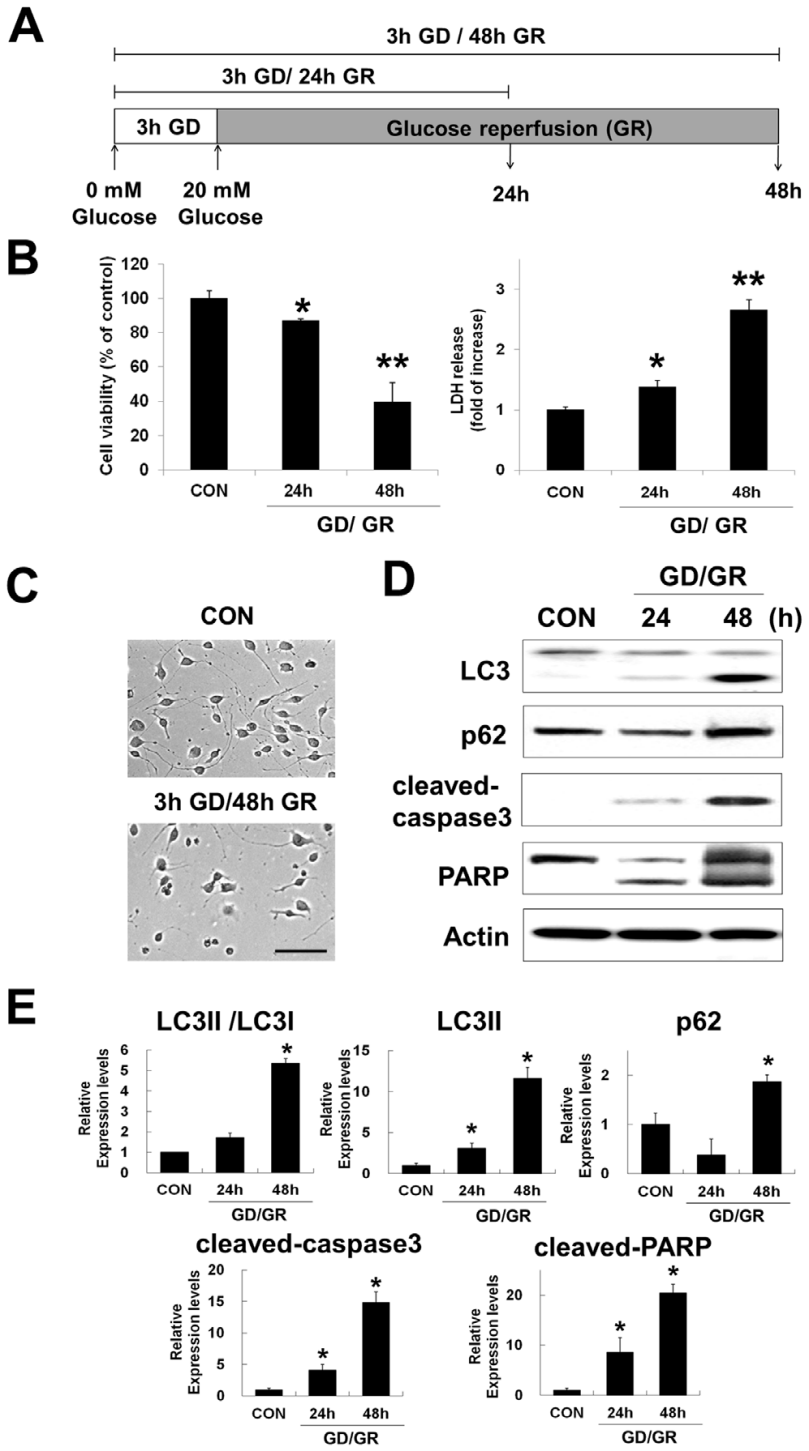
Glucose reperfusion (GR) induces further neuronal cell death. (A) Schematic diagram of experiment for the glucose reperfusion. Cells were incubated in glucose-free medium for 3 h and then replaced by 20 mM glucose containing medium for 24 h and 48 h (GD/GR). (B) Cell viability was assessed by MTT and LDH assays, respectively. MTT mitochondrial reduction was shown as a relative percentage of the MTT values at each time point (left). Cytotoxicity was calculated as the fold increase from the LDH release in control conditions. (*p<0.05, **p<0.001, n = 5). (C) Phase-contrast images of neuro2a cells after 3 h GD followed by 48 h GR (3 h GD/48 h GR). Scale bar represents 100 *µ*m. (D) The levels of LC3, p62, cleaved caspase3 and cleaved PARP were determined by immunoblot analysis. Actin was used as loading control. (E) The graphs represent relative band density of each protein shown in (D). The amount of each protein was normalized against the amount of actin. Data represent the mean ± SD (*p<0.05, n = 3).

Previously, we suggested that hypoglycemia induced-neuronal death is not due to glucose deprivation (GD) per se, but rather is a multi-factorial event initiated by glucose reperfusion (GR) [5]. To understand the cell death mechanism enacted via glucose reperfusion, cell culture conditions were established to study GR-induced neuronal death. Cultured cells were incubated in glucose free media for 3 h, followed by GR for 24 h and 48 h in 20 mM glucose containing media (Fig. 2A). The period of GD was designated as the time sufficient to reduce cell viability to approximately 50%, which was determined to be 3 hours (Fig. 1A and B). Interestingly, cell viability was significantly decreased by GR for 48 h compared to GR for 24 h [indicated values represented by mean %: 100 (control), 87.2 (24 h), 39.6 (48 h)] (Fig. 2B, left). LDH release was also significantly increased in this condition [indicated values represented by fold of increase: 1 (control), 1.38 (24 h), 2.65 (48 h)] (Fig. 2B, right). Cell death was morphologically apparent at 48 h GR following 3h GD (Fig. 2C).

To determine whether autophagic flux was affected by GD/GR, we measured levels of LC3 and p62 using immunoblot analysis (Fig. 2D). At 24 h GR after 3h GD, a slight increase in LC3II levels and a decrease in p62 – indicating activation of autophagy [10] – was observed. However, when the GR time was extended to 48 h, levels of p62 as well as LC3II and LC3II/LC3I ratio were increased, indicating a change towards inhibition of autophagic flux (Fig. 2E). At the same time, increased cell death was observed as indicated by an increase of cleaved caspase3 and cleaved PARP (Fig. 2D and E). These results indicate that inhibition of autophagic flux and activation of caspase3 are associated with GR-dependent neuronal cell death.

**Figure 3:**
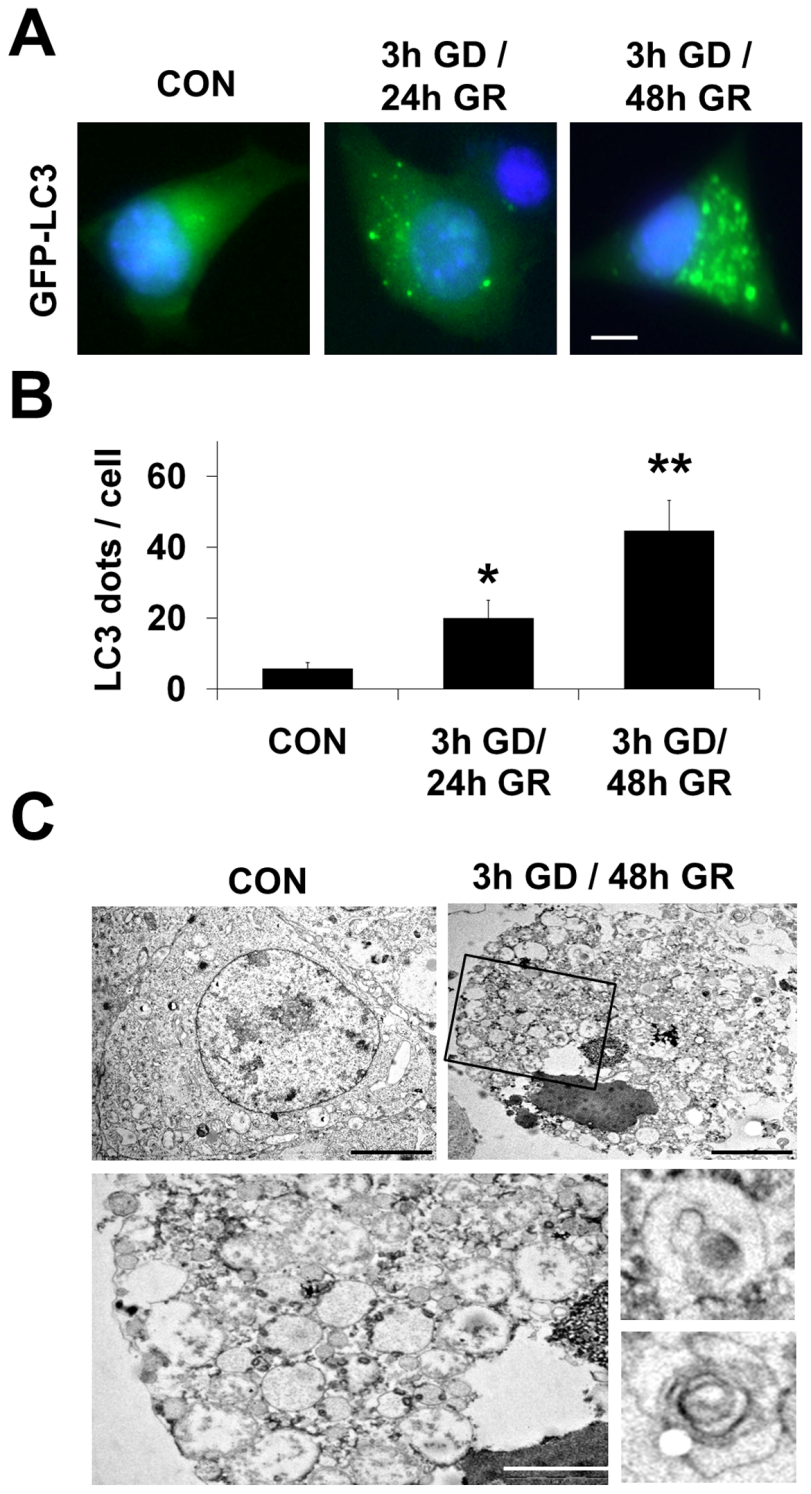
Autophagic activity is increased after glucose reperfusion (GR) in neuronal cells. (A) GFP–LC3 puncta were observed in neuro2a cells transiently transfected with the GFP–LC3 plasmid and then cultured in 3h GD followed by 24h and 48h GR at 20 mM glucose concentration. Scale bar represents 5 *µ*m. (B) Quantification of data from (A) is analyzed by mean GFP-LC3 puncta per cell (n = 20 cells per condition) from three independent experiments ± SD (*p<0.05, **p<0.001). (C) Images show ultrastructural analysis by transmission electron microscopy. Numerous autophagic vacuoles were detected in neuro2a cells treated by 3h GD followed by 48h GR. Bottom features represent enlarged autophagosomes in neuro2a after 3h GD/ 48h GR (top right, black box). Scale bars  = 5 *µ*m ; top and 1 *µ*m ; bottom.

To evaluate autophagosome formation, GFP-LC3 was transiently expressed in neuro2a cells, which were then subjected to GD for 3 h followed by two different durations of GR (24 or 48, Fig. 3A). We observed that the level of GFP-LC3 puncta in neuronal cells significantly increased according to the elongation of GR time after 3h GD (Fig. 3B). To further investigate GR-induced autophagosome formation, we observed the ultrastructural morphology of neuro2a cells using transmission electron microscopy (Fig. 3C). After 3h GD/ 48h GR, numerous autophagic vacuoles (AVs), characteristic morphological features of autophagosomes, were detected in neuro2a cells (Fig. 3C, bottom panels).

### Impairment of autophagic flux by glucose reperfusion induces activation of caspase3

**Figure 4:**
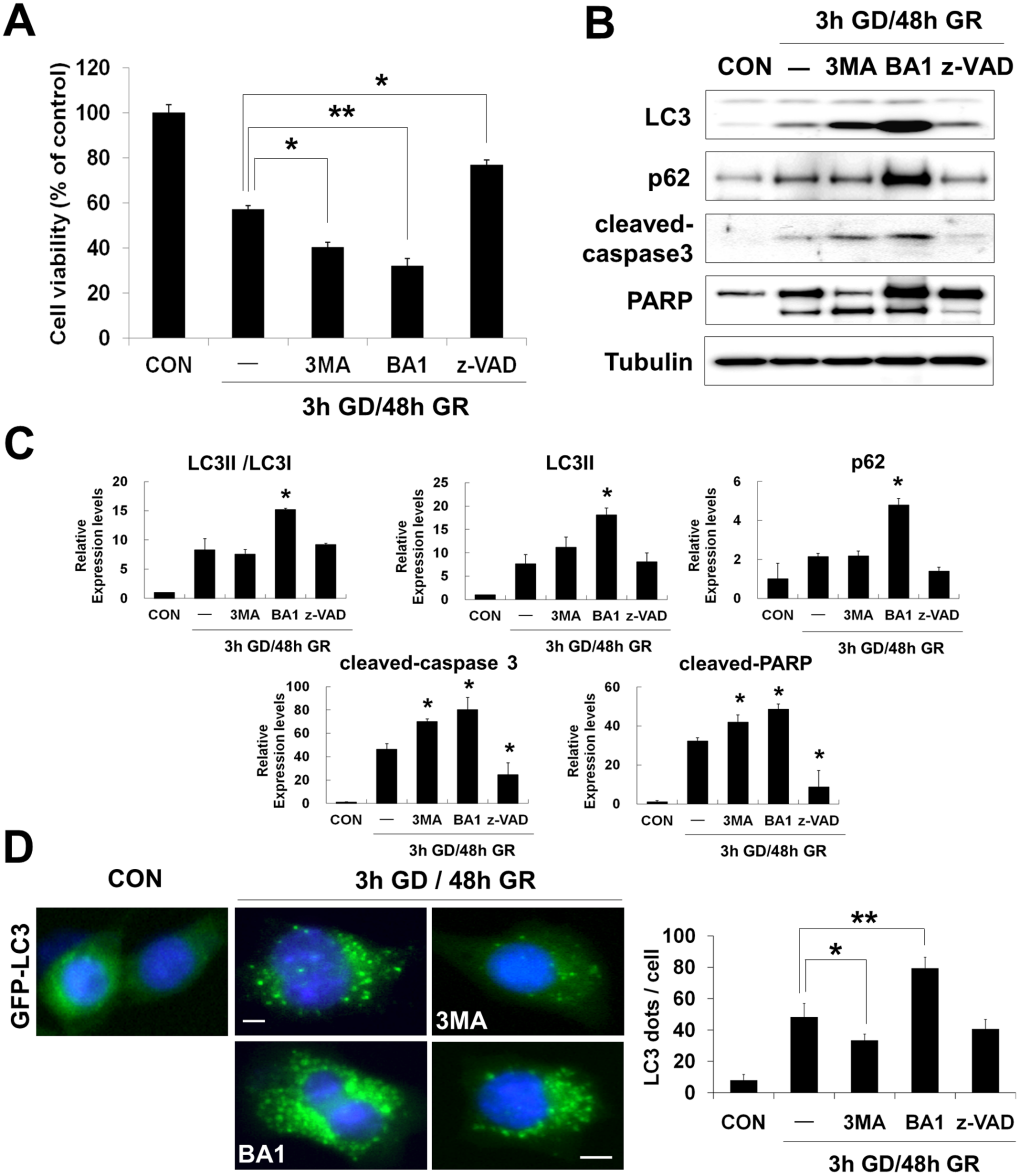
Effects of autophagy and caspase inhibitors on survival of neurons after glucose reperfusion (GR). After 3 h GD, neuro2a cells were replaced by 20-Methyladenine (3MA); Bafilomycin A1 (BA1) and z-VAD-FMK (z-VAD) were added at final concentrations of 5 mM, 5 nM and 20 uM, respectively. (A) Cell viability was assessed using the MTT assay after treatment of autophagy and pan-caspase inhibitors. MTT mitochondrial reduction was shown as a relative percentage of the MTT values at the indicated time point (3 h GD/48 h GR) from three independent experiments ± SD (*p<0.05, **p<0.001). (B) Changes in protein levels by inhibitors of autophagy or caspase activation were analyzed by immunoblot. Proteins were blotted with anti-LC3, p62, cleaved-caspase 3 and PARP antibodies. Tubulin was used as loading control. (C) Quantification of immunoblot data in (B). The amount of each protein was normalized against the amount of tubulin. Data represent the mean ± SD (*p<0.05, n = 3). (D) Cells transiently expressing GFP-LC3 (green) were treated with 3MA, BA1 or z-VAD for 48 h of GR after 3h GD. Nuclei were stained with DAPI (blue). Scale bar represents 5 *µ*m. Quantification shown on the right graph represents mean GFP-LC3 puncta per cell (n = 10 cells per condition) from three independent experiments ± SD. The asterisks (*) indicate significant differences in the values.*p<0.05, **p<0.001.

To further investigate the relationship between autophagy and apoptosis under GD/GR, effects of inhibitors on neuronal death were examined by addition of two autophagy inhibitors: 3-methyladenine (3MA) and bafilomycin A1 (BA1) were used (both of which are reported to inhibit the formation of autophagosomes or autophagolysosome) and a pan-caspase inhibitor: z-VAD-FMK (z-VAD). After 3 h GD, cells were treated with the inhibitors during the reperfusion period in 20 mM glucose containing media. After 48 h GR, cell viability was markedly decreased by 3MA (5 mM) or BA1 (5 nM) but increased by the caspase inhibitor z-VAD (20 uM), compared to GR alone [indicated values represented by mean %: 100 (control), 55.1 (GR), 40.2 (+3MA), 32.1(+BA1), 76.7(+z-VAD)] (Fig. 4A).

Next, the effects of inhibitors were analyzed by immunoblot for LC3, p62, cleaved caspase3 and PARP (Fig. 4B). Treatment of BA1 for 48 h strongly increased the accumulation of LC3II and p62 as well as caspase3 activation and PARP cleavage compared to GR alone. A similar effect on levels and activity of caspase3 was shown when the cells were exposed to 3MA during the 48 h of GR (Fig. 4C). In contrast, the caspase inhibitor z-VAD had a strong inhibitory effect on caspase3 activation, but no effect on levels of LC3II and p62, compared to GR alone (Fig. 4C). Moreover, transient overexpression of GFP-LC3 in neuro2a cells resulted in greater GD/GR-induced autophagy as indicated by higher number of LC3 puncta (Fig. 3A and 4D). This effect was blocked by the autophagosome inhibitor 3MA and further accelerated by the autolysosome inhibitor BA1 compared to GR alone (Fig. 4D). However, in cells treated with z-VAD, there was no change in levels of GFP-LC3 puncta compared to GR alone (Fig. 4D, right). In conjunction with findings from immunoblot analysis (Fig. 4B), these results suggest that following GD/GR, pharmacologic inhibition of autophagy leads to increased caspase3 activation and promotes to cell death.

### Glucose reperfusion increases expression and activity of cathepsins D and B

**Figure 5:**
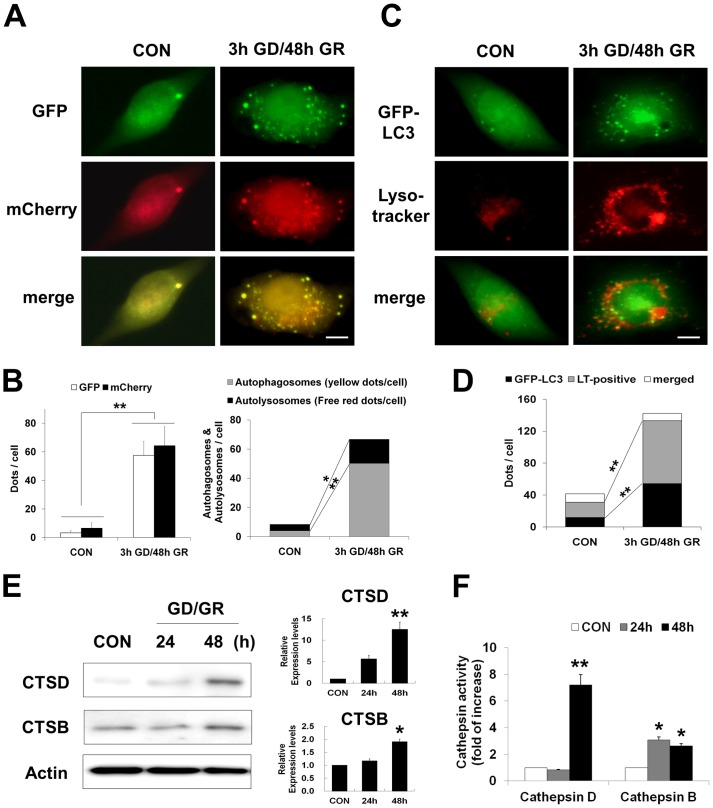
Glucose reperfusion (GR) induces cathepsin activation. (A) Representative images of fluorescent LC3 puncta. Cells were transfected with mCherry-GFP-LC3. The transfected cells were subjected to glucose deprivation for 3 h and then incubated with 20 mM glucose containing medium for 48 h (3 h GD/48 h GR). The merged images (yellow) show overlap of GFP-LC3 (green) and mCherry-LC3 (red). Scale bar represents 5 *µ*m. (B) The left graph represents the mean number of green and red puncta per cell (n = 20 cells per condition). The right graph shows the mean number of autophagosomes represented by yellow puncta in merged images and autolysosomes represented by red puncta in merged images per cell (n = 15 cells per condition) from three independent experiments ± SD (*p<0.05, **p<0.001). (C) Cells transiently expressing GFP-LC3 (green) were exposed to 3 h GD/48 h GR. Then cells were stained with LysoTracker (red). The merged images show overlap of GFP-LC3 and LysoTracker signals (yellow). Scale bar represents 5 *µ*m. (D) Quantification of data is represented from (C). Values represent mean number of GFP-LC3, positive for lysotracker (LT) stain and overlapping puncta per cell ± SD, from two independent experiments and a total of 15 cells (*p<0.05, **p<0.001). (E) Levels of cathepsin D and B were analyzed by immunoblot analysis. The neuro2a cells were harvested at the indicated time points. Actin was used as loading control. Quantification shown on the right represents relative band density of each protein from four independent experiments ± SD (*p<0.05, **p<0.001). (F) Cathepsin activity was performed at the time points indicated by cleavage of the fluorescence peptide substrate [DnP-DR-MCA, GKPILFFRLK(DnP)-DR substrate peptide labeled with MCA], as described in method. The relative activities were calculated, compared with controls and then plotted. Data represent the mean ± SD (*p<0.05, **p<0.001, n = 3).

When cells were transiently transfected with GFP-LC3, GD/GR significantly increased the number of GFP-LC3 puncta and expression levels of LC3 and p62, a protein sequestered in autophagosomes for lysosomal degradation (Fig. 3A and 2D). To separately investigate the extent of autophagosome and autolysosome activity, autophagic flux under GD/GR was assessed using mCherry-GFP-LC3, a tandem fluorescent LC3, which is labeled with acid –stable mCherry and acid-labile GFP (Fig. 5A). The numbers of GFP-LC3 puncta (green) and mCherry-LC3 puncta (red) were both significantly increased after 3h GD/48h GR compared to control (Fig. 5B, right). mCherry retains its fluorescence even in the acidic environment such as lysosome, but GFP does not. Thus, in the merged image, yellow puncta indicate autophagosome localization and free red puncta indicate autolysosome localization. The numbers of both yellow and free red puncta were increased after 3h GD/48h GR compared to control, but the magnitude of increase in autophagosome localization was greater than autolysosome localization following 3h GD/48h GR (Fig. 5B, left). Next, we monitored the intracellular localization and morphology of lysosomes using the acidotropic probe, LysoTracker (red) (Fig. 5C). Following 3h GD/48h GR, the LysoTracker signal demonstrated strong fluorescence and enlarged vesicles compared to control. Yellow puncta, which demonstrated fusion between autophagosomes and lysosomes, were observed when GFP-LC3 merged with the LysoTracker. The numbers of green (autophagosome) and red (lysosome) puncta were increased after 3h GD/48h GR compared to control, but yellow (autolysosome) puncta were rarely detected after 3h GD/48h GR (Fig. 5D). These results indicate that lysosomal dysfunction by GD/GR potentially inhibits autophagic flux at a late stage; fusion of the autophagosome and lysosome.

To confirm the presence of lysosomal dysfunction after GD/GR, we measured the levels and activities of cathepsins, major lysosomal proteases. Immunoblot analysis for cathepsin D (CatD, an aspartic protease) and cathepsin B (CatB, a cysteine protease) showed elevated immunoreactivity after GR for 48 h in 20 mM glucose concentration (Fig. 5E). Activities of CatD [indicated values represented by fold of increase: 1 (control), 0.84 (24 h), 7.21 (48 h)] and CatB [indicated values represented by fold of increase: 1 (control), 3.08 (24 h), 2.62 (48 h)] were also significantly increased after GD/GR, compared to control (Fig. 5F). These results indicated that GR leads to increase of expression and activity of cathepsin D and B.

### Excessive activation of cathepsin D and B contributes to glucose reperfusion induced neuronal death via regulation of caspase3 activity

**Figure 6:**
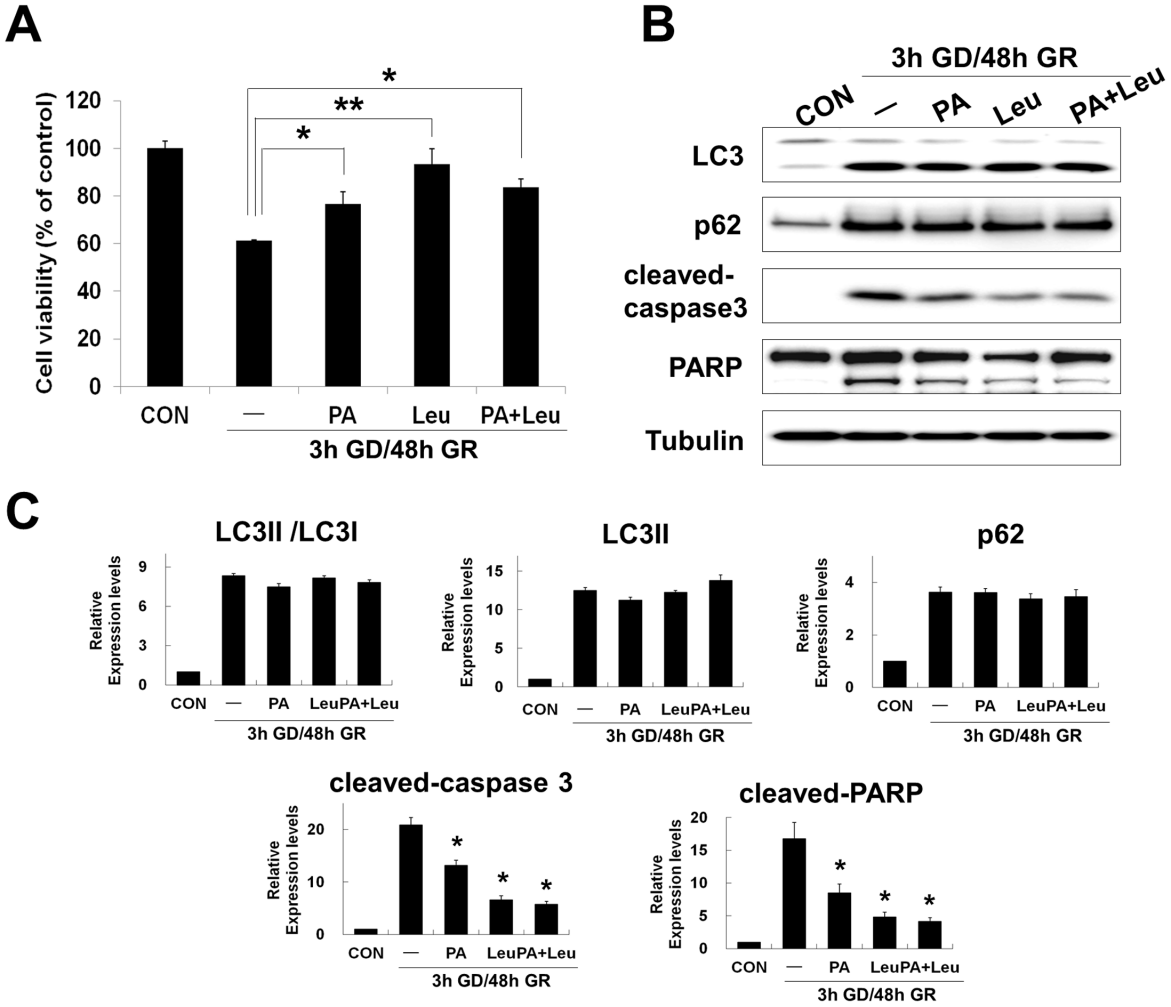
Cathepsin inhibitors promote neuronal survival after glucose reperfusion (GR). After 3 h GD, neuro2a cells were replaced by 20; pepstatin A (PA, 10 uM), leupeptin (Leu, 10 uM) or both (PA+Leu). (A) Cell viability was assessed using the MTT assay. MTT mitochondrial reduction was shown as a relative percentage of the MTT values at the indicated time point (3 h GD/48 h GR) from three independent experiments ± SD (*p<0.05, **p<0.001). (B) Changes of protein level by cathepsin inhibitors were analyzed by immunoblot analysis. Proteins from cell lysates were fractionated on SDS-PAGE; blots were probed with anti-LC3, anti-p62, anti-cleaved caspase-3, and anti-PARP antibodies. Tubulin was used as loading control. (C) The graphs represent relative band density of each protein shown in (B). The amount of each protein was normalized against the amount of tubulin. Data represent the mean ± SD (*p<0.05, n = 3).

Increased expression and activity of cathepsins have been implicated in both caspase dependent and independent cell death [28]. To examine the relationship between cathepsin activity and neuronal survival after GD/GR, we tested the effects of the following lysosomal protease inhibitors: a CatD inhibitor, pepstatin A (PA) and a CatB inhibitor, leupeprin (Leu). Treatment with CatD (PA), CatB (Leu) inhibitor or both (PA+Leu) inhibitors was sufficient to reduce GD/GR-induced neuronal death at 20 mM glucose concentration [indicated values represented by mean %: 100 (control), 61.3 (GR), 76.5 (+PA), 93.4 (+Leu), 83.6 (+PA+Leu) compared to GR alone (Fig. 6A).

To examine the relationship between lysosomal dysfunction and caspase3 activity or autophagy, we analyzed the effects of inhibitors by immunoblot for LC3, p62, cleaved caspase3 and cleaved PARP (Fig. 6B). Treatment of cathepsin inhibitors for 48 h GR resulted in reduction of cleaved-caspase3 and PARP cleavage but no change in autophagic flux as indicated by LC3II and p62 levels, compared to GR alone (Fig. 6C). These observations suggest that excessive activation of lysosomal proteases contributes to GR-induced neuronal death and may regulate neuronal death via modulation of caspase3 activity.

## Discussion

Previously, we reported that hypoglycemia-induced neuronal death does not simply arise due to glucose deprivation alone but rather due to glucose reperfusion after a certain period of glucose deprivation [29]. Conventionally, reperfusion means restoration of blood flow to ischemic tissues, which can cause additional damage of these tissues, in addition to that caused directly at the infarct site during the ischemic period [30]. This phenomenon has been demonstrated in various tissues, especially in the heart and brain, and has been termed reperfusion injury [31]. Since both hypoxia and hypoglycemia are involved in ischemia-induced tissue damage, reintroduction of oxygen, glucose or both can trigger cell death process in ischemic tissues. During reperfusion, the oxygen supplied to ischemic tissues causes an enhanced production of reactive oxygen species (ROS) thereby leading to oxidative injury of tissue [32]. Moreover, the restored blood flow magnifies the inflammatory response in damaged tissues as well as resulting in multiple processes that lead to cell death: calcium overload, excitotoxicity, ER stress and caspase-mediated apoptosis [33].

Although several mediators and mechanisms of reperfusion injury have been investigated in the ischemia/reperfusion setting, the effects of exclusively glucose-based reperfusion (GR) after hypoglycemia remain poorly understood. Accordingly, this study explored the molecular mechanism of GR-induced neuronal death after hypoglycemia. Furthermore, increasing evidence indicates that autophagy is activated during classical reperfusion injury and may be involved in the regulation of neuronal death in cell culture and animal models of ischemia/reperfusion injury in the brain [34]. Therefore, the present study focused on the role of autophagy in GR-induced neuronal death after glucose deprivation (GD) using a neuronal cell culture model. To determine whether autophagic pathways respond to glucose reperfusion (GR), two biochemical markers of autophagy were analyzed in this study: (i) the conversion of LC3I to LC3II and (ii) the protein levels of p62 [10], [35]. In neuro2a cells, GR-induced inhibition of autophagic flux was detected by increased levels of LC3II and p62 as well as increased formation LC3 puncta compared to control (Fig. 2D and 3A). This inhibition of autophagic flux by GR differed compared to activation of autophagy by glucose deprivation (GD). We found that GD lead to an increased conversion of LC3I to LC3II and a decrease in p62, which could indicate accelerated autophagic flux (Fig. 1). Recently, several studies have reported that GD induces autophagy and excessive autophagy induces cell death [36]–[38]. Although more investigation is needed to clarify which mode of cell death is engaged by both GD and GR, the difference in autophagic activity suggests GD and GR may activate divergent neuronal death mechanisms.

To investigate more specifically the role of autophagy in GR-induced neuronal death, we inhibited the autophagic flux at early stages by addition of 3MA or at late stages by addition of BA1 [10], [39]. In neuro2a cells, treatment with 3MA or BA1 led to augmented cell death compared to GR alone (Fig. 4A). In addition, our present findings that a greater accumulation of autophagic substrates (LC3II and p62) by treatment with BA1, rather than GR alone, suggests that autophagic flux is blocked at the step of autophagosome fusion with the lysosome during GR (Fig. 4B and 4D) [40]. Although further studies are needed to understand the effects of 3MA on GR-induced autophagy, induction of autophagy may play a protective role against GR-induced neuronal death in cultured neuronal cells.

In the present study, the actions of GR-induced autophagy in the neuronal cells further were confirmed by using inhibitors of the lysosomal proteases, cathepsinD and B (pepstatin and leupeptin) (Fig. 6). As mentioned above, accumulation of LC3II in the cell could be due to an increased autophagic flux or decreased autophagic degradation [35], [40]. These two possibilities can be separated by use of lysosomal protease inhibitors, and thus prevention of lysosomal degradation of autophagosomes containing LC3II and p62 [35], [41]. If the levels of higher LC3II and p62 are maintained or increased in the presence of lysosomal protease inhibitors, this indicates an increased autophagic flux. If the levels of LC3II and p62 remain unchanged, the increase in LC3II is due to failure of fusion between autophagosomes and lysosomes or degradative function of lysosomes/autolysosomes. Consistent with the results from application of autophagy inhibitors, we found no effect of lysosomal protease inhibitors on changes of LC3II and p62, indicating impaired function of the lysosome/autolysosome by GR in neuronal cells (Fig. 6B). As a consequence, pharmacological inhibition of autophagy and lysosomal proteases suggests that impaired autophagy by GR contributes to neuronal death in this setting, possibly due to autophagic stress.

Autophagic cell death or type II programmed cell death is characterized by accumulation of autophagic intermediates or extensive autophagic degradation of cellular organelles [42]. As discussed above, GR-induced neuronal death promoted accumulation of autophagosomes caused by inhibition of proteolytic steps in the autophagy pathway (Fig. 4). Impaired autophagic flux at late stages is closely related with lysosomal dysfunction. The present study found defects in the acidification of lysosomes and formation of autolysosomes after GR, which were demonstrated directly by the marked increase of LysoTracker fluorescence and merged forms of mcherry-GFP-LC3 (Fig. 5A and 5B). In addition, GR induced excessively increased expression and activation of cathepsins (Fig. 5E and 5F), which may be considered to be another marker of lysosomal dysfunction [25]. One common form of lysosomal dysfunction is lysosomal membrane permeabilization (LMP), which can lead to the release of hydrolytic enzymes that can mediate caspase-dependent apoptosis, caspase-independent apoptosis-like cell death or necrosis by massive LMP [27]. For example, the selective release of lysosomal cathepsins B or D by partial LMP may activate the mitochondrial cell death pathway. Cathepsin B or D is a major mediator of apoptotic pathways by cleavage of Bid, a proapoptotic protein which can induce mitochondrial outer membrane permeabilization (MOMP), resulting in cytochrome C release and caspase activation. Also, cathepsin D directly activates caspase [43], [44]. As expected, we found that treatment with cathepsin D/B inhibitors led to dramatically decreased levels of cleaved-caspase3, the active form of caspase3 after GR, which is in line with the observed reduction in neuronal death (Fig. 6). Thus, these results suggest that the failure of lysosomal functions induced by GR may directly affect proteolytic steps in the autophagy pathway as well as the initiation or execution steps in the caspase-dependent apoptotic pathway.

Apoptosis or type I programmed cell death is a well characterized form of programmed cell death and the sequence of molecular events involved in apoptotic death is well understood [45]. Although autophagy and apoptosis involve markedly different processes, several common pathways regulate both autophagy and apoptosis in cell death [46]. Recently, several studies have reported interconnections between the apoptotic and autophagic pathway as well as their alternative functions in cell survival or death. For example, treatment of autophagy inhibitors or knock-down/-out of autophagy-related genes (e.g. *Atg5, Atg10 and Atg12*) results in accumulation of cytoplasmic components and promotion of apoptosis [47]. On the other hand, pharmacological or genetic inhibition of autophagy prevents cell death via regulation of apoptotic pathway in cell culture models under specific conditions [48], [49]. Given this context, levels of cleaved-caspase3, one of the execution-caspases in apoptotic cell death, were increased by GR in the present study (Fig. 2D). Treatment with autophagy inhibitors, 3MA and BA1 increased caspase3-dependent apoptosis in GR-induced neuronal death (Fig. 4A and 4B). However, z-VAD had no effect on levels of autophagic substrates (LC3II and p62) in spite of its potent inhibitory effect on caspase3 activation (Fig. 4B). These observations show that inhibition of autophagy, both at early and late stages of the process, may lead to apoptosis and ultimately neuronal cell death after GR. From these results, the autophagic and the apoptotic pathways might be functionally interconnected, where enhanced apoptosis is due to impaired autophagy in GR-induced neuronal death.

Furthermore, autophagic processes interconnect with apoptosis through proteins shared by both autophagic and apoptotic pathways [50]. For example, Atg5, one of the autophagy related genes (*Atgs*) has an essential role in autophagosome formation but can also stimulate apoptotic machinery. Yousefi *et al*. reported that calpain-mediated cleavage of Atg5 promotes cytochrome C release and caspase activation thus serving as a switch between autophagy to apoptosis [51]. Another example, Beclin-1, which is a component of the type III PI3 kinase complex for initiating autophagy, interacts with members of the anti-apoptotic Bcl-2 family. Interestingly, the interaction between Bcl-2 and Beclin 1 via the BH3 domain inhibits autophagy [50]. Wirawan *et al.* reported that caspase-mediated cleavage of Beclin-1 not only inhibits autophagy but also enhances apoptosis by promoting the release of proapoptotic factors from mitochondria such as Bak and Bok [52]. By contrast, unlike other BH3-only proteins (pro-apoptotic proteins), high levels of Beclin-1 does not disturb the protective effect of Bcl-2 against apoptosis despite the binding of Bcl-2 to Beclin-1 [53]. In the present study, inhibition of autophagy modulated the activation of caspase3 while z-VAD does not affect autophagy in GR-induced neuronal cell death (Fig. 4). Therefore, autophagy may be an upstream event of caspase activation in this setting. However, many questions remain regarding how autophagy regulates apoptosis and which proteins act as molecular switches between autophagy and apoptosis in GR-induced neuronal death.

Taken together, the present study shows that impaired autophagy after GR in neuronal cells was demonstrated by a failure to degrade autophagic substrates and to clear AVs as a result of a specific defect in degradation of lysosomes/autolysosomes. In addition, the present study demonstrates: i) abnormal activities of autophagy and caspase3 after GR, ii) LC3II accumulation or caspase3 activation by autophagy or cathepsin inhibitors in GR-induced neuronal death, and iii) excessive expression and activities of cathepsin B and D after GR in cultured neuronal cells. Therefore, this study proposes that an interplay of autophagy, apoptosis and the lysosome is a major contributing source of GR-induced neuronal death. Furthermore, the present study suggests that induction of well-controlled autophagy or suppression of inappropriate autophagy may have neuroprotective effects against glucose reperfusion after severe hypoglycemia in the brain.
